# Myo1b Promotes Premature Endothelial Senescence and Dysfunction via Suppressing Autophagy: Implications for Vascular Aging

**DOI:** 10.1155/2023/4654083

**Published:** 2023-01-09

**Authors:** Yi Yu, Yuanyuan Ren, Zi Li, Yang Li, Yirong Li, Yan Zhang, Runlin Gui, Yue Cui, Lu Qian, Yuyan Xiong

**Affiliations:** ^1^Key Laboratory of Resource Biology and Biotechnology in Western China, Ministry of Education, Faculty of Life Sciences and Medicine, Northwest University, Xi'an, Shaanxi, China; ^2^Department of Endocrinology, Xi'an No.3 Hospital, The Affiliated Hospital of Northwest University, Xi'an, Shaanxi, China; ^3^Xi'an Key Laboratory of Cardiovascular and Cerebrovascular Diseases, Xi'an No.3 Hospital, The Affiliated Hospital of Northwest University, Northwest University, Xi'an, Shaanxi, China

## Abstract

Endothelial cell (EC) senescence characterized by an irreversible growth arrest leading to endothelial dysfunction has been implicated in vascular aging and aging-associated cardiovascular diseases. Autophagy plays a crucial role in the modulation of cellular senescence. Our previous showed that myosin 1b (Myo1b), one family of nonfilamentous class-1 myosin, was reported to be involved in the modulation of human smooth muscle cell senescence. However, the role of Myo1b in the modulation of EC senescence with links to autophagy has yet to be elucidated. In this study, we sought to explore the role of Myo1b in endothelial senescence and further elucidate the underlying mechanisms. Here, we show prominent upregulation of Myo1b in senescent ECs in comparison with nonsenescence ECs in both mRNA and protein expression levels. Silencing Myo1b in senescent cells ameliorates endothelial dysfunctions and reverses endothelial senescence phenotypic changes such as senescence-associated-*β*-galactosidase activity, cyclin-dependent kinase inhibitor p21^WAF1^, expression of vascular adhesion molecule-1 (VCAM1) and intercellular adhesion molecule-1 (ICAM1), and the senescence-associated cytokines. In contrast, in nonsenescent cells, overexpressing Myo1b promotes endothelial senescence and suppresses autophagy through the impairment of autophagosome and lysosome fusion. The interaction between Myo1b and LRRK2 through Myo1b tail domain promotes intracellular calcium elevation, which results in the inhibition of autophagic flux. *In vitro* and *in vivo* aging models, Myo1b knockdown in senescent ECs and wild type-aged mice is able to enhance autophagy and ameliorate aging-associated endothelial dysfunction. Taken together, our studies reveal a new function for Myo1b, that is, to couple LRRK2 assembly to promote an increase in intracellular calcium level, which impairs the autophagosome-lysosome fusion, and ultimately the promotion of EC senescence and vascular aging.

## 1. Introduction

Aging, a major risk factor for cardiovascular diseases, is progressively associated with vascular complications due to premature cellular senescence, proinflammation, and dysregulation in endothelium-derived vasoactive molecules [1]. Accumulating evidence reveals that endothelial cell senescence, an irreversible and stable growth arrest in response to various triggers, results in the vascular tone regulation disturbance and endothelial dysfunction, ultimately contributing to vascular aging and age-associated vascular disorders [2, 3]. Senescent endothelial phenotypes are featured by increased senescence-associated-*β*-galactosidase (SA-*β*-gal) activity, overproduced superoxide anion, reduced vasodilation molecule (nitric oxide, NO) production, accumulated DNA damage, accompanied by elevated adhesion molecules expression such as vascular adhesion molecule-1 (VCAM), and intercellular adhesion molecule-1 (ICAM1) [3, 4]. The increase in endothelial adhesion molecule expression leads to the recruitment of monocytes from the circulation and transendothelial migration into vascular wall, which crucially influences the initiation and development of age-accelerated atherogenesis [5, 6]. Moreover, endothelial cell (EC) senescence tends to secret a variety of senescence-associated inflammatory cytokines and/or chemokines such as TNF-*α*, IL-6, and IL-8, which is termed senescence-associated secretory phenotype (SASP), and can impair the function of neighboring cells or EC selves [1, 7].

The mechanisms of cellular senescence are multifactorial, of which, autophagy has been demonstrated to play a role in the regulation of cellular senescence [8]. It refers to a set of processes that engulf intracellular proteins or damaged organelles and wrap them into vesicles, and then fuse with lysosomes to form autophagosome-lysosomes to degrade the encapsulated contents [9]. This process is not only capable of preventing the accumulation of cell wastes, but also recycling autophagy-degraded substances as nutrients for cell life activities under energy-deficient conditions [10]. Numerous proteins are involved in these processes, of which, the protein microtubule-associated protein 1 light chain 3 (LC3) and the adaptor protein SQSTM1/p62 are commonly used to monitor autophagic degradation/flux [11]. Accumulation of LC3 and SQSTM1 reflects defective autophagic flux and degradation, whereas low SQSTM1 levels may indicate active degradation. The autophagosome-lysosome fusion, the core regulator of autophagic degradation/flux, depends on a variety of different factors including the internal acidity, ATG8 family proteins, autophagy-related SNARE proteins, and also the intracellular calcium [12, 13]. Autophagosome-lysosome fusion was defective in bafilomycin A1-treated or Ca-P60A/dSERCA-depleted cells with a high concentration of intracellular Ca^2+^ [14]. However, the identification of proteins regulating Ca^2+^-dependent vesicle fusion still remains elusive.

Although the role of autophagy in EC senescence is not completely understood, emerging studies suggest that impaired autophagy is closely associated with cardiovascular function decline and increased susceptibility to cardiovascular disease upon aging [15]. Adequate boost of autophagy protects against cellular senescence and maintains cellular homeostasis in endothelial and smooth muscle cells, resulting in antiatherosclerotic effects [11, 16].

Myosin 1b (Myo1b) belongs to one family of nonfilamentous class-1 myosin that comprises an N-terminal motor domain containing the ATP and actin-binding sites, a light chain-binding neck region known as an IQ domain, and a C-terminal tail domain containing a pleckstrin homology (PH) domain [17, 18]. Growing evidence indicates the important role of Myo1b in the modulation of various cellular functions, including cell proliferation and apoptosis [19, 20]. In cervical cancer (CC), Myo1b was reported to promote cell proliferation, migration, and invasion through the activation of c-MYC, in turn contributing to cervical carcinogenesis and tumor development [19]. In the cellular myocardial ischemia/reperfusion (I/R) injury model, upon I/R stimulation, Myo1b expression was reduced, and overexpressing Myo1b prevented the hypoxia/reoxygenation-induced cardiomyocytes apoptosis and proliferation inhibition in H9c2 cells [21]. Notably, our previous study demonstrated that Myo1b was implicated in the activation of ARG2-mTORC1 signaling axis that promotes smooth muscle cell senescence and apoptosis [18]. Next, we further uncover that Myo1b interacting with PTEN modulates nuclear AKT activation and cell apoptosis via blockading the nuclear localization of PTEN in melanoma cells B16-F10 [22]. mTORC1, AKT, and PTEN play essential roles in the modulation of cell senescence and proliferation [23–25], which strongly promotes us to hypothesize the causal connections between Myo1b and endothelial cell senescence/proliferation. Given that impaired autophagy is implicated in cellular senescence and vascular aging, thus, in this study, we investigated the role of Myo1b and the potential mechanism in the regulation of EC senescence with links to autophagy.

## 2. Materials and Methods

### 2.1. Materials

Reagents were purchased or obtained from the following sources: anti-myosin 1b antibody (ab194356, Abcam); anti-VCAM-1 antibody (sc-13160, Santa Cruz Technology); anti-*λ*H2AX antibody (sc-517348, Santa Cruz Technology); ICAM-1 antibody (sc-8439, Santa Cruz Technology); anti-p21antibody (sc-6246, Santa Cruz Technology); anti-LC3A/B antibody (#4108S, Cell Signaling Technology); anti-LAMP1 antibody (#9091S, Cell Signaling Technology); anti-p62 antibody (18420-1-AP, Proteintech); anti-LRRK2 antibody (#5559S, Cell Signaling Technology); and anti-tubulin antibody (SAB4500087, Sigma-Aldrich). Duolink® In Situ Detection Reagents Red (DUO92008) was from Sigma. IRDye 800-conjugated affinity purified goat anti-rabbit IgG F(c) was purchased from LI-COR Biosciences (Lincoln, Nebraska, USA); goat anti-mouse IgG (H+L) secondary antibody Alexa Fluor® 680 conjugate, goat anti-mouse IgG (H+L) secondary antibody Alexa Fluor® 488 conjugate, goat anti-rabbit IgG (H+L) secondary Antibody Alexa Fluor® 488 conjugate, and goat anti-rabbit IgG (H+L) secondary antibody Alexa Fluor® 594 conjugate were from Invitrogen/Thermo Fisher Scientific (Waltham, MA, USA). Insulin-transferrin-selenite sodium and dexamethasone were from Sigma (St. Louis, Missouri, USA). All cell culture media and materials were purchased from Gibco/Thermo Fisher Scientific (Waltham, Massachusetts, USA).

### 2.2. Generation and Purification of Recombinant Adenovirus (rAd)

Generation of rAd-expressing shRNA-targeting human or mouse Myo1b driven by the U6 promoter (rAd/U6-hMyo1b shRNA or rAd/U6-mMyo1b shRNA) was carried out with the Gateway Technology. The targeting sequences are indicated in underline below [18]:

hMyo1b-shRNA:

5′-CACCGGGCTTTATGGATCATGAAGCCGAAGCTTCATGATCCATAAAGCCC-3′.

mMyo1b-shRNA:

5′-CACCGGAGCTCCTCTACAAGCTTAACGAATTAAGCTTGTAGAGGAGCTCC-3′.

rAd/U6-LacZ shRNA served as control was generated as previously described [18]. The purification and mouse tail vein injection of rAd/U6-mMyo1b shRNA adenovirus particles were performed as described by Tan et al. [26]. Generation of rAd expressing myc-MYO1B-WT and its mutants -R165A (deficient in its motor activity) and -K966A (deficient in its C-terminal PH domain) driven by CMV promoter (rAd/CMV-myc-MYO1B-WT, -R165A, and -K966A) was also carried out with the Gateway Technology. The expression plasmids encoding myc-MYO1B-WT, -R165A, and-K966A were kindly provided by Lynne M. Coluccio [17].

### 2.3. Animals

The wild-type female young (2–3 months old) and old (23–24 months old) C57BL/6J were obtained from Cyagen Biosciences (Suzhou, China). The mice had free access to food and water and were maintained in a room with controlled humidity (50%) and temperature (22°C~25°C) on a 12 h light/dark cycle. The old mice were subjected to tail vein injection with either purified adenovirus- (Ad-) mediated scramble shRNA or sh Myo1b for the knockdown of Myo1b (200 mL/mice); each injection interval is two days, and the total injection lasts for 20 days. Then, mice were anesthetized with xylazine (10 mg/kg (body weight), intraperitoneally) and ketamine (100 mg/kg (body weight), intraperitoneally) and sacrificed. Thoracic aortas were isolated and cleaned from perivascular fat and subjected to *en face* staining or snap frozen directly in liquid nitrogen and kept at -80°C till further immunoblotting analysis. All animal experiments were approved by the Animal Ethics Committee of Northwest University (no. NWU-AWC-20220202M) and performed in accordance with the Association for Assessment and Accreditation of Laboratory Animal Care guidelines.

### 2.4. Cell Culture and Adenoviral Transduction

Human umbilical vein endothelial cells (HUVECs) were prepared and maintained in RPMI-1640 medium supplemented with 5% FCS and ECGS [27]. Nonsenescent endothelial cells with low passage (P2-P3) are referred to as “young” cells. For preparation of senescent cells, the “young” cells were further split continuously till replicative senescence as evaluated by SA-*β*-gal staining [4]. Cell transduction with the recombinant adenovirus for gene silencing or overexpression was performed as previously described [3]. Human monocytic cell line THP-1 was cultured in RPMI-1640 containing 10% heat-inactivated fetal bovine serum (HIFBS).

### 2.5. Senescence-Associated *β*-Galactosidase (SA-*β*-Gal) Staining

SA-*β*-gal staining was performed as previously described [11]. Briefly, cells were washed twice with PBS followed by fixation with 4% formaldehyde solution in PBS for 10 min at room temperature. Wash the fixed cells twice with PBS, cells were then incubated with the SA-*β*-gal staining solution (1 mg/mL X-gal, 40 mmol/L citric acid, 5 mmol/L potassium ferrocyanide, 5 mmol/L potassium ferricyanide, 150 mmol/L sodium chloride, 2 mmol/L magnesium chloride dissolved in phosphate buffer, and pH 6.0) overnight at 37°C in a CO_2_-free atmosphere. Following the incubation, wash the cells for twice with PBS and once with methanol and allow them to air dry. The stained senescent cells were viewed and documented by conventional microscopy.

### 2.6. Measurement of Intracellular Calcium (Ca^2+^)

For the detection of intracellular Ca^2+^, experimental HUVECs were gently washed twice with PBS, and incubated with 5 *μ*m Fura-2 AM in PBS at 37°C for 30 minutes. After the incubation, wash cells three times with PBS. Images were documented by fluorescence microscopy (Nexcope NIB900) with excitation at 340 nm. Quantification of the signals was performed using NIH Image 1.62 software.

### 2.7. Immunoblotting

Cell lysates were prepared by lysing cells in lysis buffer (120 mM NaCl, 50 mM Tris (pH 8.0), 20 mM NaF, 1 mM benzamidine, 1 mM EDTA, 1 mM EGTA, 1 mM sodium pyrophosphate, 30 mM 4-nitrophenyl phosphate disodium salt hexahydrate, 1% NP-40, and 0.1 M phenylmethylsulfonyl fluoride (PMSF)). Next, 40 *μ*g extracts were subjected to sodium dodecyl sulfate-polyacrylamide gel electrophoresis and electrophoretically transferred to an Immobilon-P membrane (Millipore), and the resultant membrane was incubated overnight with the corresponding primary antibody at 4°C with gentle agitation after being blocked with 5% skimmed milk (Yepuri et al., 2012) [3]. The blot was then further incubated with a corresponding anti-mouse (Alexa Fluor 680 conjugated) or anti-rabbit (IRDye 800 conjugated) secondary antibody. Signals were visualized using Odyssey Infrared Imaging System (LI-COR Biosciences). Quantification of the signals was performed using NIH Image 1.62 software (U. S. National Institutes of Health).

### 2.8. In Situ Proximity Ligation Assay (PLA)

HUVECs cultured on coverslips were washed with PBS, and then incubated in ice cold 100% methanol for 10 minutes at -20°C, rinse in PBS for 5 minutes, permeabilized in 0.3% Triton X-100 for 10 min, and blocked with Duolink Blocking Solution. After blocking, cells were incubated with mixed anti-Myo1b and anti-LRRK2 primary antibodies overnight at 4°C. Cells were then incubated with the PLUS and MINUS PLA probes diluted 1 : 5 in the Duolink Antibody Diluent in a preheated humidified chamber for 1 h at 37°C. Subsequent ligation, amplification, and detection were performed according to manufacturer's instructions. Fluorescence images were acquired using a Leica TCS SP5 confocal laser microscope and the signals of PLA were quantified with NIH Image 1.62 software (U. S. National Institutes of Health).

### 2.9. Immunoprecipitations

HUVEC cells growing in 10 cm dishes were rinsed once with cold PBS and lysed on ice for 20 min in 1 mL of ice-cold lysis buffer (40 mM HEPES (pH 7.5), 120 mM NaCl, 1 mM EDTA, 10 mM pyrophosphate, 10 mM glycerophosphate, 50 mM NaF, and EDTA-free protease and phosphatase inhibitors) containing 0.3% CHAPS. After centrifugation at 13,000 × g for 10 min, the protein concentration of cleared supernatant was measured by the DC™ Protein Assay (5000112, Bio-Rad). 5 *μ*g of the indicated antibodies was added to the 800 *μ*g protein supernatant and incubated with rotation overnight at 4°C. 20 *μ*L of Protein A/G PLUS-Agarose (sc-2003, Santa Cruz Biotechnology) was then added and the incubation continued for 2 h at room temperature. Pulled-down immunoprecipitates were then washed three times with lysis buffer. Samples were resolved by SDS-PAGE and proteins were transferred to PVDF and visualized by immunoblotting.

### 2.10. Immunofluorescence Staining

Cells cultured on glass coverslips were fixed with 4% paraformaldehyde for 15 min at room temperature and then permeabilized with 0.2% Triton X-100 and blocked with 1% BSA in PBS for 60 mins. Coverslips were incubated with the corresponding primary antibody overnight at 4°C, followed by incubation with Alexa Fluor-labeled secondary antibodies for 2 h at room temperature, and mounted. Images were acquired through 40x objectives with Leica TCS SP5 confocal laser microscope. Representative images taken at the same exposure and magnification are shown in all figures.

### 2.11. Quantitative Real-Time Reverse Transcription PCR (qRT-PCR) Analysis

Total RNA was extracted from cells with TRIzol Reagent (Thermo Fisher Scientific Inc.) following the supplier's protocol. First-strand cDNA was synthesized from 500 ng total RNA with GoScript™ Reverse Transcriptase and random primers (A2801, Promega). Real-time PCR was performed with the GoTaq® qPCR (A6001, Promega) and CFX Connect Real-Time PCR Detection System (Bio-Rad). mRNA expressions were normalized to the reference gene glyceraldehyde 3-phosphate dehydrogenase (GAPDH). PCR primers are as follows:

human Myo1b-1F: CTCCTACAGCAGGCTCACAGTT.

human Myo1b -1R: GCCTCGTTGAAGATGTGTGCTG.

human GAPDH-F: TGCACCACCAACTGCTTAGC.

human GAPDH-R: GGCATGGACTGTGGTCATGAG.

### 2.12. Enzyme-Linked Immunosorbent Assay (ELISA)

The protein level of secreted cytokines for IL6, IL8, TNF-*α*, and MCP-1 in the conditioned medium was collected after serum starvation for overnight and determined with the use of the ELISA MAX Deluxe from BioLegend according to manufacturer's instruction.

### 2.13. Detection of NO and Superoxide Level in Endothelial Cells

NO and superoxide levels in endothelial cells were measured by staining the cells with fluorescent dyes DAF-2DA and DHE, respectively, as described previously [3]. Briefly, for detection of NO, HUVECs were gently washed twice with Ca^2+^-free PBS and incubated in a modified Krebs-Ringer bicarbonate buffer containing 118 mmol/L NaCl, 4.7 mmol/L KCl, 2.5 mmol/L CaCl_2_, 1.2 mmol/L MgSO_4_, 1.2 mmol/L KH_2_PO_4_, 25 mmol/L NaHCO_3_, 0.026 mmol/L EDTA, and 5.5 mmol/L glucose mixed with 5 mmol/L of DAF-2DA for 30 minutes. For measurement of cytoplasmic superoxide, cells were incubated with 5 mmol/L DHE dissolved in culture medium for 30 minutes. The cells were then washed 3 times and images were obtained with fluorescence microscopy. Quantification of the signals was quantified by NIH ImageJ software.

### 2.14. Flow Cytometry

Briefly, cells at a seeding density of 2 × 10^5^cells/well were seeded in a six-well plate; for detection of NO, HUVECs were gently washed twice with Ca^2+^-free PBS, and incubated with 10 *μ*mol/L of DAF-2DA for 30 minutes at 37°C in the dark. For measurement of cytoplasmic superoxide, cells were incubated with 10 *μ*mol/L DCFH-DA dissolved in culture medium for 30 minutes at 37°C in the dark. Then, the cells were collected following trypsin digestion and suspended in 0.5 mL PBS. Measurements were carried out using flow cytometry at 488 nm excitation wavelength and using a 520 and 550 nm emission band-pass filter. A minimum of 30000 events per sample was analyzed. Data processing was performed using FlowJo software version 10.8.1 for Windows.

### 2.15. *En Face* Detection of NO and Superoxide in Intact Mouse Aortas

The NO and superoxide anion generation in mouse aorta were evaluated by DAF-2DA and DHE staining, respectively, as previously described [28]. Briefly, young (2–3 months) and old mice (23–24 months) mice aortas without perivascular tissues were equilibrated for 30 minutes in Krebs buffer at 37°C aerated with 95% O_2_ and 5% CO_2_. After equilibration, aortas were incubated with 5 *μ*mol/L DAF-2DA or 5 *μ*mol/L DHE for 30 minutes or 10 minutes, respectively. Wash three times with Krebs buffer and fixed in 4% paraformaldehyde followed by staining with DAPI (300 nmol/L, 3 minutes). Wash the aortas three times with PBS and carefully cut them longitudinally and mount *en face* (face down) on slides with mounting medium for endothelial layer imaging. The images from DAF-2DA, DHE, and DAPI staining were quantified with ImageJ software and results are presented as the ratio of DAF-2DA and DAPI positive nucleus or ratio of DHE and DAPI. The fluorescence images were captured by the Leica SP8 confocal microscopy.

### 2.16. Monocyte Adhesion to Endothelial Cells

For monocyte-endothelial cell adhesion assays [3], THP-1 cells were incubated with 5 *μ*mol/L CFDA-SE in PBS at 37°C for 8 minutes. After adding 1 mL of heat-inactivated FBS for 1 minute to stop the labeling, 4 × 10^5^-labeled THP-1 were then added to the HUVECs to incubate for 15 min at 37°C. After washing the nonadherent THP-1 cells twice with PBS, HUVECs were fixed in 4% paraformaldehyde. The images of adherent monocytes were viewed under the fluorescent microscope. The number of adherent monocytes was counted using the NIH ImageJ software (U. S. National Institutes of Health).

### 2.17. Statistics

Data are given as mean ± SEM. In all experiments, *n* represents the number of experiments. Statistical analysis was performed with unpaired *t*-test or ANOVA with the Dunnett or the Bonferroni posttest. Differences in mean values were considered significant at *p* < 0.05.

## 3. Results

### 3.1. Upregulated Myo1b and SASP in Senescent Endothelial Cells Are Accompanied with Endothelial Dysfunction

In our previous study, we revealed that Myo1b serves as a mediator in arginase-II- (ARG2-) induced smooth muscle cell senescence through positively regulating mTORC1-S6K1 signaling cascade [18]. Here, we showed that, as the human endothelial cells were undergoing senescence (S) evaluated by positively stained for SA-*β*-gal as the cellular senescence marker (Supplementary Material online, Figure S1A), both of the protein and mRNA levels of Myo1b were significantly augmented in comparison with the to the low-passage cells, referred to as “young” (Y) cells (Supplementary Material online, Figure S1B-C). Moreover, enhanced intracellular superoxide (DHE), decreased NO (DAF-2DA), and increased THP-1 monocyte-HUVEC adhesion, as the featured hallmarks of endothelial dysfunction, were also observed in the senescent endothelial cells (Supplementary Material online, Figures S1D-E). The SASP of endothelial cells was also examined by ELISA; as compared to young cells, prominent elevation of a variety of extracellular inflammatory cytokines such as IL-6, IL-8, TNF-*α*, and MCP-1 was observed in senescent HUVECs **(**Supplementary Material online, Figure S1F**)**.

### 3.2. Silencing Myo1b in Senescent Cells Reverses Endothelial Senescent Phenotypic Changes and Ameliorates Endothelial Functions

**Figure 1:**
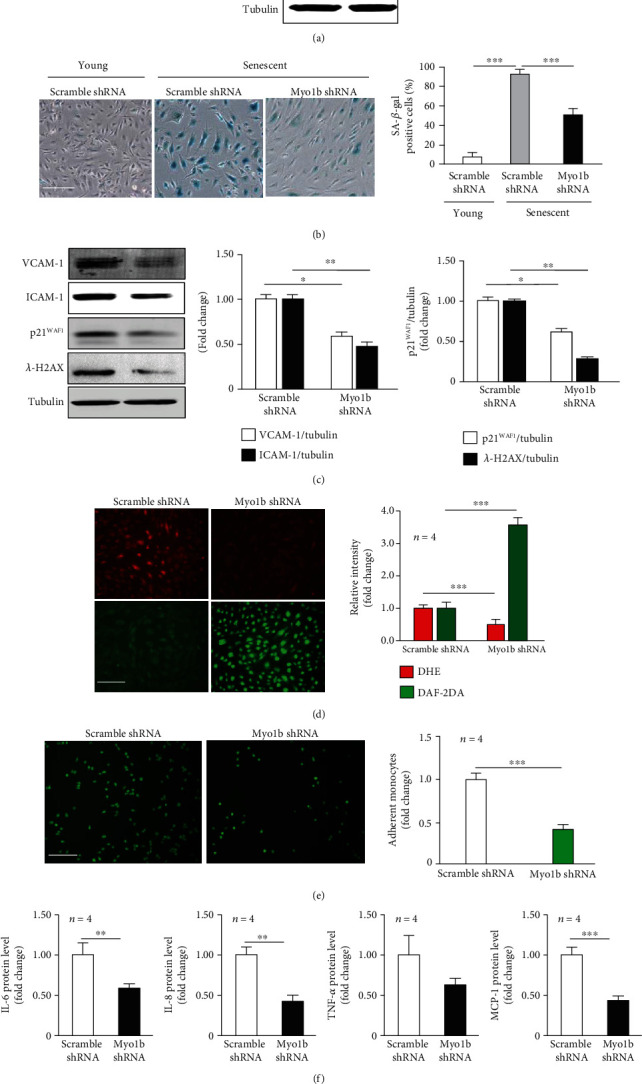
Silencing Myo1b in senescent cells attenuates endothelial senescent phenotypic changes, dysfunctions, and inflammations. Young and senescent human umbilical vein endothelial cells (HUVECs) were transduced with rAd/U6-scramble shRNA as control and rAd/U6-Myo1b shRNA for silencing Myo1b. After 3 days of transduction and 16 h of serum starvation, (a) immunoblotting confirms silencing efficiency in senescent cells. (b) SA-*β*-gal staining of each group. Bar graphs show quantifications of positive cells. Scale bar = 0.25 mm. (c) Immunoblotting analysis of senescence markers p21 levels, vascular adhesion molecule-1 (VCAM-1), and intercellular adhesion molecule-1 (ICAM-1) and DNA damage marker *λ*-H2AX expression. Bar graphs show quantifications of the markers. Tubulin served as loading control. (d) DHE staining for detection of superoxide anion and DAF-2DA staining for detection of NO. Quantifications of DHE and DAF-2DA signals are shown on the right panel. (e) THP-1 monocyte-HUVEC adhesion analysis. Bar graphs show quantifications of the adhered monocytes. (f) The secretion of IL-6, IL-8, TNF-*α*, and MCP-1 was determined by ELISA with collected conditioned medium. (*n* = 4, ^∗^ indicates *p* < 0.05, ^∗∗^ indicates *p* < 0.01, ^∗∗∗^ indicates *p* < 0.001).

We then found that the depletion of Myo1b in senescent HUVECs as validated by immunoblotting (Figure 1(a)) remarkably reduced the number of SA-*β*-gal positive cells (Figure 1(b)). Immunoblotting analysis also showed that silencing Myo1b resulted in the significant decrease in the expression of adhesion molecules VCAM-1, ICAM-1, cell cycle inhibitor p21^WAF1^, and DNA damage marker *γ*-H2AX (Figure 1(c)). Moreover, senescence-induced superoxide overproduction, NO reduction, and monocyte-HUVEC adhesion were dampened in Myo1b-deficient senescent endothelial cells (Figures 1(d) and 1(e)). In addition, silencing Myo1b was able to reduce the secretion a variety of extracellular inflammatory cytokines such as IL-6, IL-8, TNF-*α*, and MCP-1 in senescent endothelial cells (Figure 1(f)), suggesting the curial role of Myo1b in the modulation of endothelial senescence, endothelial function, and senescence-associated inflammation.

### 3.3. Overexpressing Myo1b Promotes Endothelial Senescence and Dysfunction

**Figure 2:**
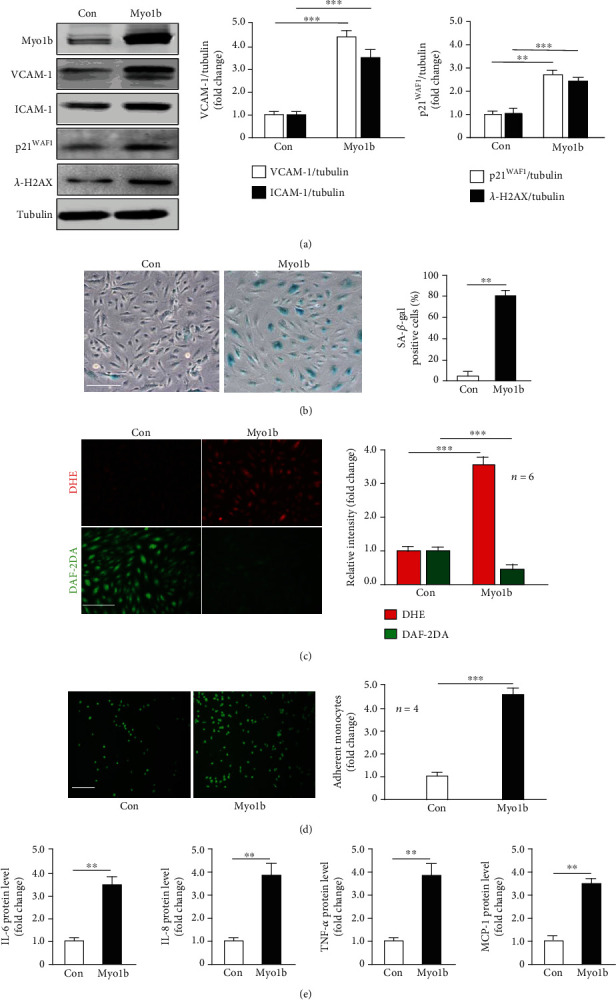
Myo1b overexpression induces nonsenescent EC senescence and dysfunctions. Young HUVECs were transduced with rAd/CMV as control and -MYO1B for overexpressing. After 3 days of transduction and 16 h of serum starvation, (a) immunoblotting shows the effects of Myo1b overexpression on p21, VCAM-1, ICAM-1, and *λ*-H2AX. Bar graphs show quantifications of the markers. Tubulin served as loading control. (b) SA-*β*-gal staining. Bar graphs show quantifications of positive cells. Scale bar = 0.25 mm. (c) DHE and DAF-2DA staining for the detection of superoxide anion and NO. Quantifications of these signals are shown on the right panel. (d) Monocyte-HUVEC adhesion analysis. Bar graphs show quantifications of the adhered monocytes. (e) ELISA analysis of the secretion of IL-6, IL-8, TNF-*α*, and MCP-1 from conditioned medium. (*n* = 4, ^∗∗^ indicates *p* < 0.01, ^∗∗∗^ indicates *p* < 0.001).

Next, in young HUVECs, recombinant adenovirus-mediated ectopic expressing a wild-type Myo1b as confirmed by immunoblotting promoted the expression levels of VCAM-1, ICAM-1, *γ*-H2AX, and cell cycle inhibitor p21^WAF1^ (Figure 2(a)). Meanwhile, upon the overexpression of Myo1b, the young HUVECs tended to accelerate the senescence evaluated by the augmented number of SA-*β*-gal positive cells (Figure 2(b)) and the occurrence of endothelial dysfunctions, e.g., the impaired NO production (DAF-2DA) and enhanced intracellular superoxide production (Figure 2(c)). As expected, due to the prosenescent effect of Myo1b, the young HUVECs transduced with the recombinant adenovirus expressing Myo1b significantly exacerbated the capacity of monocyte-HUVEC adhesion (Figure 2(d)), as well as the proinflammatory cytokines releases of IL-6, IL-8, TNF-*α*, and MCP-1 (Figure 2(e)).

### 3.4. Myo1b Promotes Endothelial Cell Senescence and Dysfunction via Suppressing Autophagy

**Figure 3:**
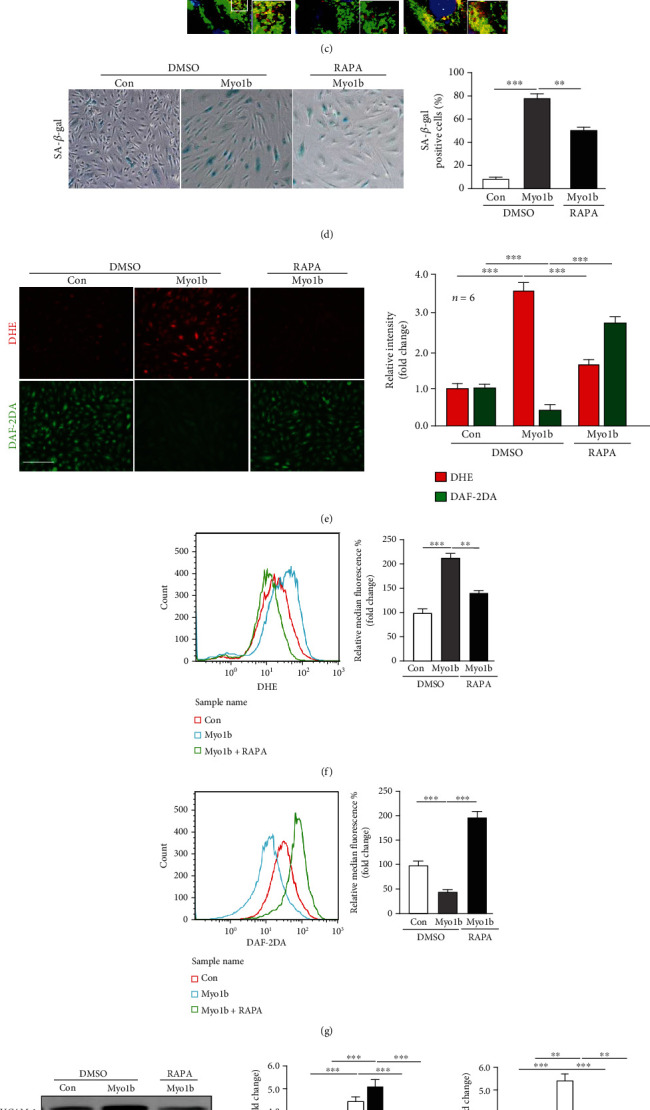
Myo1b promotes endothelial cell senescence through the inhibition of autophagy. Young HUVECs were transduced as in Figure 2(a); cell lysates were prepared and subjected to (a) immunoblotting analysis of Myo1b, LC3-I/-II, SQSTM1/p62, and *λ*-H2AX without or with Baf A1 (20 nmol/L) treatment. Quantification of the signals is also shown. Tubulin served as loading control. (b) The immunofluorescence staining for LC3-I/-II (red) and DAPI (blue), and (c) for LC3-I/-II (red), lysosome marker LAMP1 (green), and DAPI (blue) of Myo1b overexpressed HUVECs without or with rapamycin (20 *μ*m). The enlargements of selected area are shown on the lower left. Shown are representative merged images from 4 independent experiments. Scale bar = 25 *μ*m. (d) SA-*β*-gal staining. Bar graphs show quantifications of positive cells. Scale bar = 0.25 mm. (e) DHE and DAF-2DA staining for the detection of superoxide anion and NO. Flow cytometry analysis of intracellular superoxide anion (f) and NO levels (g). Quantifications of these signals are shown on the right panel. (h) The immunoblotting analysis of Myo1b, LC3-I/-II, SQSTM1/p62, p21, and *λ*-H2AX without or with rapamycin (20 *μ*m). (*n* = 4, ^∗∗^ indicates *p* < 0.01, ^∗∗∗^ indicates *p* < 0.001).

Substantial evidences highlighted that autophagy played a key role in the modulation of cellular senescence and several types of myosin were implicated with autophagy regulation [11, 16, 29–31], which prompted us to investigate whether Myo1b promotes endothelial cell senescence and dysfunction through mediating autophagy. Intriguingly, immunoblotting analysis showed that overexpression of Myo1b in young HUVECs significantly boosted the accumulation of LC3-II and autophagy adaptor protein p62, which cannot be observed in the group of bafilomycin A1 (Baf A1, 20 nmol/L, 60 min), a vacuolar H^+^-ATPase (V-ATPase) inhibitor that blocks the fusion of autophagosome-lysosomes (Figure 3(a)). Confocal microscopic immunofluorescence staining also showed the increased LC3 puncta area upon Myo1b overexpression (Figure 3(b)). These results indicate that the upregulation of Myo1b expression in endothelial cells can suppress the autophagosome-lysosomes fusion and degradation of LC-II and p62, which was confirmed by the coimmunostaining of autophagosomes (LC3) and lysosomes (LAMP1) (Figure 3(c)). We further explored whether the restoration of autophagic flux is able to prevent senescence-promoting effects of Myo1b in young HUVECs. For this purpose, low concentration of rapamycin [32–34] was utilized to restore the autophagosome-lysosome fusion in Myo1b-overexpressed young HUVECs (Figure 3(c)). Interestingly, we observed that the senescence (SA-*β*-gal-stained positive cells) (Figure 3(d)) and endothelial dysfunction, i.e., overproduced superoxide and reduced NO levels (Figure 3(e)), provoked by Myo1b were significantly reversed by rapamycin. Flow cytometry shows consistent results that rapamycin protects against the increase in superoxide (Figure 3(f)) and decrease in NO generation (Figure 3(g)) in young HUVECs with Myo1b overexpression. In addition, elevated VCAM1, ICAM1, cell cycle inhibitor p21^WAF1^, and DNA damage marker *γ*-H2AX expression were also prevented by rapamycin (Figure 3(h)). These results suggested that Myo1b mediates endothelial autophagy through interfering with the autophagic flux, which modulates the endothelial senescence and senescence-associated endothelial dysfunction.

### 3.5. Myo1b Negatively Regulates Autophagy by Suppressing the Autophagosome-Lysosome Fusion through Its Pleckstrin Homology (PH) Domain

**Figure 4:**
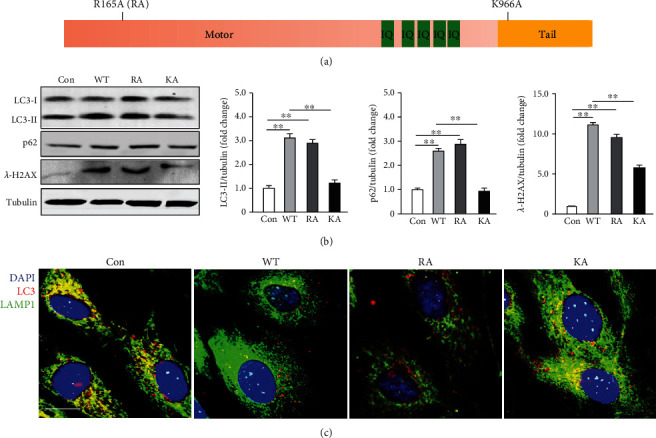
Myo1b suppresses the autophagosome-lysosome fusion. (a) The domain structure of Myo1b and mutants. Young HUVECs were transduced as in Figure 2(a); young HUVECs were transduced with rAd/CMV as control, rAd/CMV-myc-MYO1B-WT (WT), rAd/CMV-myc-MYO1B-R165A (RA), and rAd/CMV-myc-MYO1B-K966A (KA) for overexpression, (b) immunoblotting analysis of LC3-I/-II, SQSTM1/p62, and and *λ*-H2AX, quantification of the signals is shown on the right panel, and tubulin served as loading control. (*n* = 4, ^∗∗^ indicates *p* < 0.01). (c) The immunofluorescence staining for LC3-I/-II (red), lysosome marker LAMP1 (green), and DAPI (blue). Shown are representative merged images from 4 independent experiments. Scale bar = 25 *μ*m.

The full length of Myo1b is composed of a motor domain, a neck domain with five IQ domains, and a tail domain (Figure 4(a)). Next, to determine which domain of Myo1b is responsible for the suppressive effects on autophagosome-lysosome fusion, two Myo1b mutants, R165A (a mutant in the motor domain) and K966A (a mutant in its C-terminal PH domain) were constructed (Figure 4(a)) and verified in our previous study [22]. Intriguingly, only R165A but not K966A exhibited similar function with WT in the accumulation of LC3 and p62, as well as impaired autophagosome-lysosome fusion. In compassion with WT group, immunoblotting analysis showed K966A mutant but not R165A mutant remarkably reduced the accumulation of LC3, p62, and *γ*-H2AX (Figure 4(b)) and did not impair the autophagosome-lysosome fusion (Figure 4(c)), indicating that the tail of domain of Myo1b is the crucial component to mediate Myo1b induced autophagy suppression in endothelial cells.

### 3.6. Myo1b Interacts with LRRK2 to Promote Intracellular Ca^2+^ Elevation

**Figure 5:**
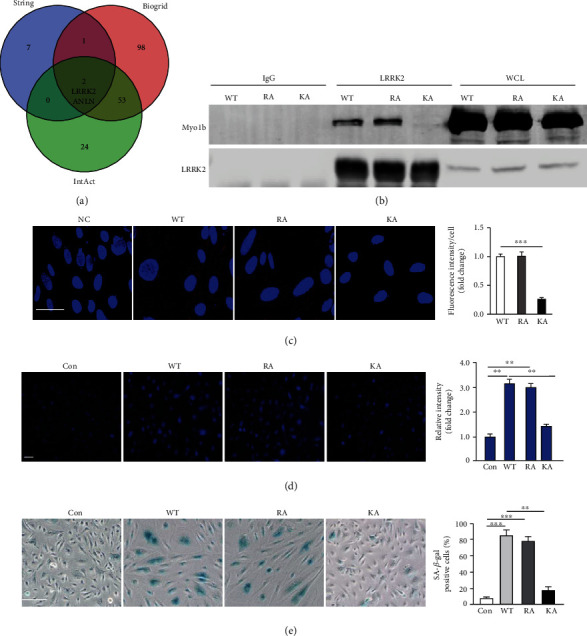
Myo1b interacts with LRRK2 to regulate intracellular Ca^2+^ level. (a) Venn diagram-based analysis of Myo1b-protein interactions from STRING, BioGRID, and IntAct. Young HUVECs were transduced with rAd/CMV as control (con), rAd/CMV-myc-MYO1B-WT (WT), rAd/CMV-myc-MYO1B-R165A (RA), and rAd/CMV-myc-Myo1b-K966A (KA) for overexpression, (b) immunoblotting analysis of Myc-Myo1B and LRRK2 in the whole-cell lysates (WCL), and immunoprecipitates using an anti-Myo1b antibody was performed. Immunoprecipitation using a normal IgG served as negative control. (c) Duolink PLA for protein interaction between LRRK2 and Myo1b in MEF cells with the overexpression of WT, RA, and KA (cells without primary antibody as the negative control (NC)). Scale bar = 50 *μ*m. (d) Detection of intracellular calcium by Fura-2 AM staining. (5E) SA-*β*-gal staining. Bar graphs show quantifications of positive cells. Scale bar = 0.25 mm. Quantification of the signals is also shown. Scale bar = 100 *μ*m. (*n* = 4, ^∗∗^ indicates *p* < 0.01, ^∗∗∗^ indicates *p* < 0.001).

Protein-protein interaction (PPI) acts as a crucial regulator in many essential cellular functions and biological processes [35]. By scanning the commonly used PPI database, two candidates, leucine-rich repeat serine/threonine-protein kinase 2 (LRRK2) and aniline (ANLN), were screened out in the Venn diagram of Myo1b-binding proteins (Figure 5(a)). Of which, LRRK2 has been reported to be implicated in a variety of cellular processes including autophagy [36]. Coimmunoprecipitated (Co-IP) validated the interaction between Myo1b and LRRK2, and further showed that K966A mutant lost the capability for interacting with LRRK2 in the nonsenescent HUVECs (Figure 5(b)), suggesting that Myo1b tail part containing the pleckstrin homology (PH) domain is indispensable for this interaction. Moreover, this result was further verified by proximity ligation assay (PLA) (Figure 5(c)). In addition, we found that both WT and R165A mutant, but not K966A, promoted the prominent elevation of intracellular Ca^2+^ and senescence in young HUVECs (Figures 5(d) and 5(e)). Collectively, these results demonstrate that Myo1b tail domain interacting with LRRK2 impairs endothelial autophagy through promoting intracellular Ca^2+^ level elevation tail domain, ultimately contributing to cellular senescence.

### 3.7. Silencing Myo1b Strengthens Autophagy in Senescent Endothelial Cells

**Figure 6:**
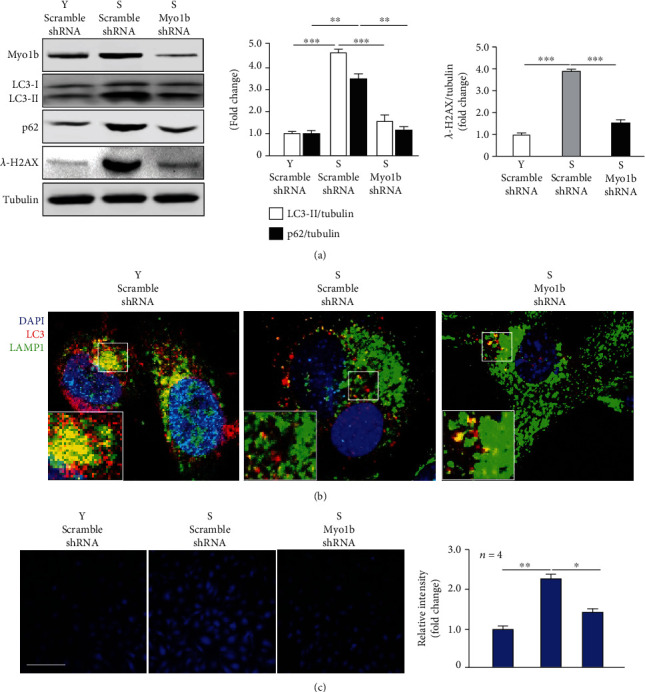
Silencing Myo1b promotes autophagy in senescent endothelial cells. Senescent HUVECs were transduced with rAd/U6-LacZ-short hairpin RNA (shRNA) as control and rAd/U6-Myo1b shRNA for silencing Myo1b. After 3 days of transduction and 16 h of serum starvation, cells are subjected to (a) the immunoblotting analysis of Myo1b, LC3-I/-II, SQSTM1/p62, and *λ*-H2AX; (b) the immunofluorescence staining for LC3-I/-II (red), lysosome marker LAMP1 (green), and DAPI (blue); the enlargements of selected area are shown on the lower left. (c) The detection of intracellular calcium by Fura-2 AM staining. Quantification of the signals is also shown. Y: young HUVECs; S: senescent HUVECs; scale bar = 100 *μ*m. (*n* = 4, ^∗^ indicates *p* < 0.05, ^∗∗^ indicates *p* < 0.01, ^∗∗∗^ indicates *p* < 0.001).

In senescent HUVECs, endogenous Myo1b is significantly elevated with concomitant suppressive autophagy evaluated by enhanced LC3, p62, and *γ*-H2AX as compared to the young cells (Figure 6(a)). Interestingly, this senescence-associated endothelial autophagy inhibition was attenuated by the depletion of Myo1b (Figure 6(a)). Confocal immunostaining showed that the impairment of autophagosome-lysosome fusion was observed in senescent HUVECs, which was prevented by silencing Myo1b (Figure 6(b)). Furthermore, increased Ca^2+^ in senescent cells was also prevented upon silencing Myo1b (Figure 6(c)), supporting the finding that Myo1b enhancing intracellular Ca^2+^ level leads to the inhibition of endothelial autophagy.

### 3.8. Myo1b Knockdown in Mice Ameliorates Aging-Associated Endothelial Dysfunction

**Figure 7:**
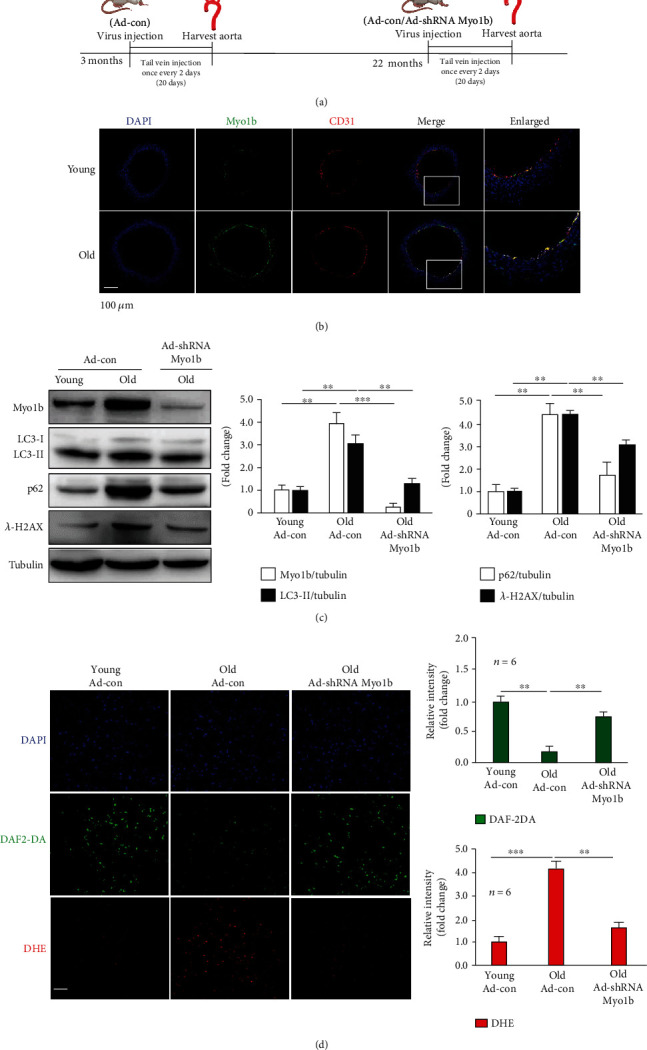
Myo1b knockdown in mice ameliorates aging-associated endothelial dysfunction. (a) Experimental timeline of virus administration and aorta harvest time points of young (3-4 months) and old (23-24 months) mice. Aortas isolated from each group of mice were subjected to (b) immunofluorescence costaining of Myo1b with endothelial marker CD31 in cryosection biopsy. Old WT mice are injected, respectively, with purified adenovirus- (Ad-) mediated expression of scramble shRNA as control and sh Myo1b for the knockdown of Myo1b. Aortas were isolated and subjected to (c) immunoblotting analysis of Myo1b, LC3-I/-II, SQSTM1/p62, and *λ*-H2AX; tubulin is taken as loading control. (d) *En face* DHE (for detection of superoxide anion) and DAF-2DA (for detection of NO) followed by counter staining with DAPI of the aortas. Scale bar = 100 *μ*m. (*n* = 6, ^∗∗^ indicates *p* < 0.01, ^∗∗∗^ indicates *p* < 0.001).

The experimental timeline of virus administration and tissue harvest for each replicate of young and old mice was indicated in Figure 7(a). In *in vivo* mice model, we further showed the Myo1b (Myo1b, green signal) protein expression in the aortic endothelial cells (CD31, red signal) of old mice was markedly upregulated as compared to the young animals (Figure 7(b)). Also, autophagy suppression evaluated by increased LC3-II, p62, and *γ*-H2AX was also observed in the aorta of old mice (Figure 7(c)). Immunoblotting analysis confirmed the efficient gene knockdown of Myo1b by tail injection of adenovirus expressing shRNA targeting mouse Myo1b in the aorta of old mice (Figure 7(c)). In line with cellular findings, adenovirus-mediated knockdown of Myo1b in old mice significantly ablated the aging-induced suppression assessed by the reduction of LC3-II, p62, and *γ*-H2AX (Figure 7(c)). Also, the decrease in NO production (DAF-2DA) and increase in ROS production (DHE) in the aortas of aged mice were blockaded by Myo1b knockdown (Figure 7(d)).

## 4. Discussion

EC senescence has been considered a crucial event in the occurrence of cardiovascular diseases such as atherosclerosis [37]. It is associated with aging-related vascular dysfunction that is characterized by increased superoxide anion, decreased NO production, elevated inflammatory cytokines, and adhesion molecule expression [3], whereas, the molecular mechanisms of EC senescence and related vascular impairment in aging are still not well elucidated. In this study, we, for the first time, identified that Myo1b as a novel regulator of EC senescence mediates endothelial dysfunction with a link to vascular aging through the suppression of endothelial autophagy.

Our previous study reveals that Myo1b is involved in the activation of ARG2-mTORC1-S6K1 signaling axis that promotes vascular smooth muscle cell senescence [18]. Herein, we further investigated the role of Myo1b in the regulation of EC senescence linked to vascular impairment in aging. Our results firstly confirm that, with the EC senescence and mouse vascular aging, Myo1b in protein and gene expression levels is significantly enhanced in the senescent human ECs and vascular endothelium of aortas of aged WT mice as compared to young cells and young WT mice, respectively. Furthermore, Myo1b gene knockdown in senescent ECs and old mice remarkably attenuates cellular senescence with concomitant restoration of endothelial function, and reduction of senescence-associated secretory phenotype (SASP) characterized by the decreased of proinflammatory cytokines releases, suggesting that Myo1b plays a crucial role in the regulation endothelial aging and aging-associated endothelial dysfunction. This conclusion is further reinforced by experiments of overexpressing Myo1b in young ECs showing that it significantly drives young cell senescence and endothelial dysfunction and SASP. Overall, the results from the cultured cells and mouse model provide both in vitro and in vivo firm evidence for a causal role of Myo1b in promoting endothelial inflammation, endothelial dysfunction, and vascular aging. The role of myosin in the modulation of cellular senescence was still rarely investigated. Previously, it has been reported that nonmuscle myosin regulatory light chain (nmMLC20) could induce endothelial progenitor cell (EPC) senescence and dysfunctions in pulmonary artery hypertension (PAH) rat model through the upregulation of NADPH oxidase- (NOX-) derived reactive oxygen species [38]. In human nucleus pulposus cells, myosin IIA and IIB interacting with actin regulated compression stress-induced human NP cell senescence via the activation of RhoA/ROCK1 pathway [39]. Thus, myosin, beyond its function as motor protein, might serve as a novel character in the modulation of cellular senescence.

How does Myo1b drive human EC senescence? Numerous studies demonstrate that autophagy as an essential intercellular process for degrading unfolded proteins aggregates and damages organelles through the lysosomal machinery plays a pivotal role in the regulation of cellular senescence [31, 40]. Here, we find that Myo1b promoting young EC senescence, endothelial dysfunction, and inflammation is accompanied by the impairment of the autophagosome-lysosome fusion, which is reversed by the rapamycin that promotes autophagosome-lysosome fusion. Importantly, the suppression of autophagic flux is also observed in the replicative senescence ECs with the high expression of Myo1b. Silencing Myo1b in these senescent ECs not only restores the senescence-associated autophagic flux but also improves the senescence-associated endothelial dysfunction. In addition, Myo1b gene knockdown mice model further confirms our cellular findings. Indeed, Myo1b knockdown in the old mice significantly improves the aging-induced endothelial dysfunction with the concomitant elevation of vascular autophagy, suggesting the causal role of Myo1b-associated autophagy impairment in cardiovascular aging *in vivo*. Recently, Lee et al. show that the inhibition of autophagy by palmitic acid (PA) promotes EC senescence, and the recovery of autophagy and autophagic flux attenuates PA-induced endothelial senescence [16]. Moreover, in endothelial progenitor cells (EPCs), autophagy level decreased with the occurrence of senescence, and enhancing the autophagy by expressing lncRNA-p21 remarkably attenuated the EPC senescence [41]. In accordance, it is conceivable that autophagy presents a crucial regulatory mechanism in the modulation of EC senescence and vascular aging.

In our current study, we, for the first time, disclose the inhibitory effects of Myo1b on the fusion between autophagosome and lysosome. In HeLa cells, a previous study shows that myosin VI (Myo VI), an actin-based motor protein, promotes the autophagosome maturation and fusion with the lysosome through binding with its adaptor partners on endosomes [42]. Myosin 1C (Myo1C), another subtype of myosin, has been shown that the loss of its motor may alter the lipid composition of autophagic, leading to the disruption of autophagosome-lysosome fusion in HeLa cells [43]. Myo1b has been found in the endosomes in MNT1 cells [44]; however, in ECs, additional experiments are required to investigate whether it mediates the delivery of endocytic cargo to autophagosomes (Myo VI) or lipid composition of autophagic (Myo1C). However, by mutating the motor (R165A) and tail (K966A) domains of Myo1b, results from both immunofluorescence staining and immunoblotting confirm that the loss of myo1b tail domain containing PH domain is not able to inhibit autophagic flux in ECs, suggesting that Myo1b, unlikely Myo VI and Myo1C, may mediate the autophagic flux independently of its motor property. Taken together, accumulating evidence support an emerging concept that different myosins with diverse structural and biochemical properties present distinct functions and molecular mechanisms.

Another important novel finding of this study is that Myo1b positively regulates the intracellular calcium level in vascular aging. Intercellular calcium serves as a vital regulator in a variety of cellular processes including cellular senescence and autophagy [45, 46], which is supported by our results that EC senescence blocks autophagy and enhances intracellular calcium level. In our study, significant Ca^2+^ elevation is observed in young ECs overexpressing Myo1b or senescent ECs with upregulated Myo1b, which prompts us to propose for the first time that Myo1b is capable of facilitating the elevation of intracellular calcium level, which further result in the inhibition of autophagosome-lysosome fusion. Currently, for researchers, the role of calcium in the regulation of autophagy still remains controversial, which depends on the cell types and circumstances [47]. In ECs, our study provides evidence that enhanced cytosolic calcium suppresses autophagy through impairing the autophagic flux, which is consistent with the finding that elevation of Ca^2+^ induced by cadmium inhibits the autophagosome-lysosome fusion to block the degradation of autophagosomes in primary rat proximal tubular (rPT) cells [48].

Previous studies indicated that binding sites existed within the C-terminal tail region of Myo1b containing a putative PH domain [17]. By means of searching protein-protein interaction databases, LRRK2 is identified to be conjugated with Myo1b to promote the elevation of intracellular calcium in ECs. In agreement with several reports that LRRK2 plays a vital role in intracellular calcium homeostasis through different mechanisms including facilitating calcium extrusion exchanger and acidic store alterations [49, 50], CO-IP analysis further reveals that the PH domain contained in Myo1b tail part is the functional domain for binding with LRRK2, which is consistent with the results that PH domain mutant lacks the suppressive effects of WT Myo1b on autophagosome-lysosome fusion and intracellular calcium increase. A similar finding has been reported to show that LRRK2 regulates autophagy through a calcium-dependent protein kinase-*β* (CaMKK-*β*)/adenosine monophosphate- (AMP-) activated protein kinase (AMPK) pathway [51], whereas, additional investigation is required to elucidate the specific molecular mechanism of Myo1b-LRRK2 interaction modulating intracellular calcium concentration. Coincidentally, earlier studies show that LRRK2 is a key influencing factor for Parkinson disease (PD), which is significant to be associated with aging [52], indicating that Myo1b might be involved in the pathogenies of PD through mediating the neural cell senescence. Altogether, these results demonstrate that Myo1b conjugating with LRRK2 through its PH domain promotes the increase of intracellular calcium, which suppresses the autophagosome-lysosome fusion, ultimately leading to cellular senescence and aging.

## 5. Conclusion

In summary, our current work reveals a new function for Myo1b, that is, to couple LRRK2 assembly to promote intracellular calcium increase, which results in the impairment of autophagosome-lysosome fusion, and ultimately, the promotion of EC senescence and vascular aging. This represents another novel mechanism of that Myo1b regulates endothelial aging and a promising therapeutic approach to target vascular aging and age-associated cardiovascular diseases.

## Data Availability

The datasets used and/or analyzed during the current study are available from the corresponding authors on reasonable request.

## Supplementary Materials

Supplementary Materials Supplementary Figure 1: upregulated Myo1b and cytokine secretion in senescent endothelial cells with endothelial dysfunction. (A) SA-*β*-gal staining of young and senescent HUVECs. (B) The protein and (C) mRNA levels of Myo1b in senescent endothelial cells. Bar graphs show quantifications of the markers. Tubulin served as loading control. (D) DHE and DAF-2DA staining for the detection of superoxide anion and NO. (E) THP-1 monocyte-HUVECs adhesion analysis. Bar graphs show quantifications of the adhered monocytes. (F) ELISA analysis of the secretion of IL-6, IL-8, TNF-*α*, and MCP-1 from conditioned medium. Scale bar = 0.25 mm. Y: young HUVECs; S: senescent HUVECs; *n* = 4, ^∗^ indicates *p* < 0.05, ^∗∗^ indicates *p* < 0.01, ^∗∗∗^ indicates *p* < 0.001).
