# Supplementary material for: Myo1b Promotes Premature Endothelial Senescence and Dysfunction via Suppressing Autophagy: Implications for Vascular Aging
Source: Oxid Med Cell Longev. 2023 Jan 9;2023:4654083. doi: 10.1155/2023/4654083 (PMC9842418; doi:10.1155/2023/4654083)
Supplement: Supplementary Materials — Supplementary Figure 1: upregulated Myo1b and cytokine secretion in senescent endothelial cells with endothelial dysfunction. (A) SA-β-gal staining of young and senescent HUVECs. (B) The protein and (C) mRNA levels of Myo1b in senescent endothelial cells. Bar graphs show quantifications of the markers. Tubulin served as loading control. (D) DHE and DAF-2DA staining for the detection of superoxide anion and NO. (E) THP-1 monocyte-HUVECs adhesion analysis. Bar graphs show quantifications of the adhered monocytes. (F) ELISA analysis of the secretion of IL-6, IL-8, TNF-α, and MCP-1 from conditioned medium. Scale bar = 0.25 mm. Y: young HUVECs; S: senescent HUVECs; n = 4, ∗ indicates p < 0.05, ∗∗ indicates p < 0.01, ∗∗∗ indicates p < 0.001). [file 4654083.f1.pdf]

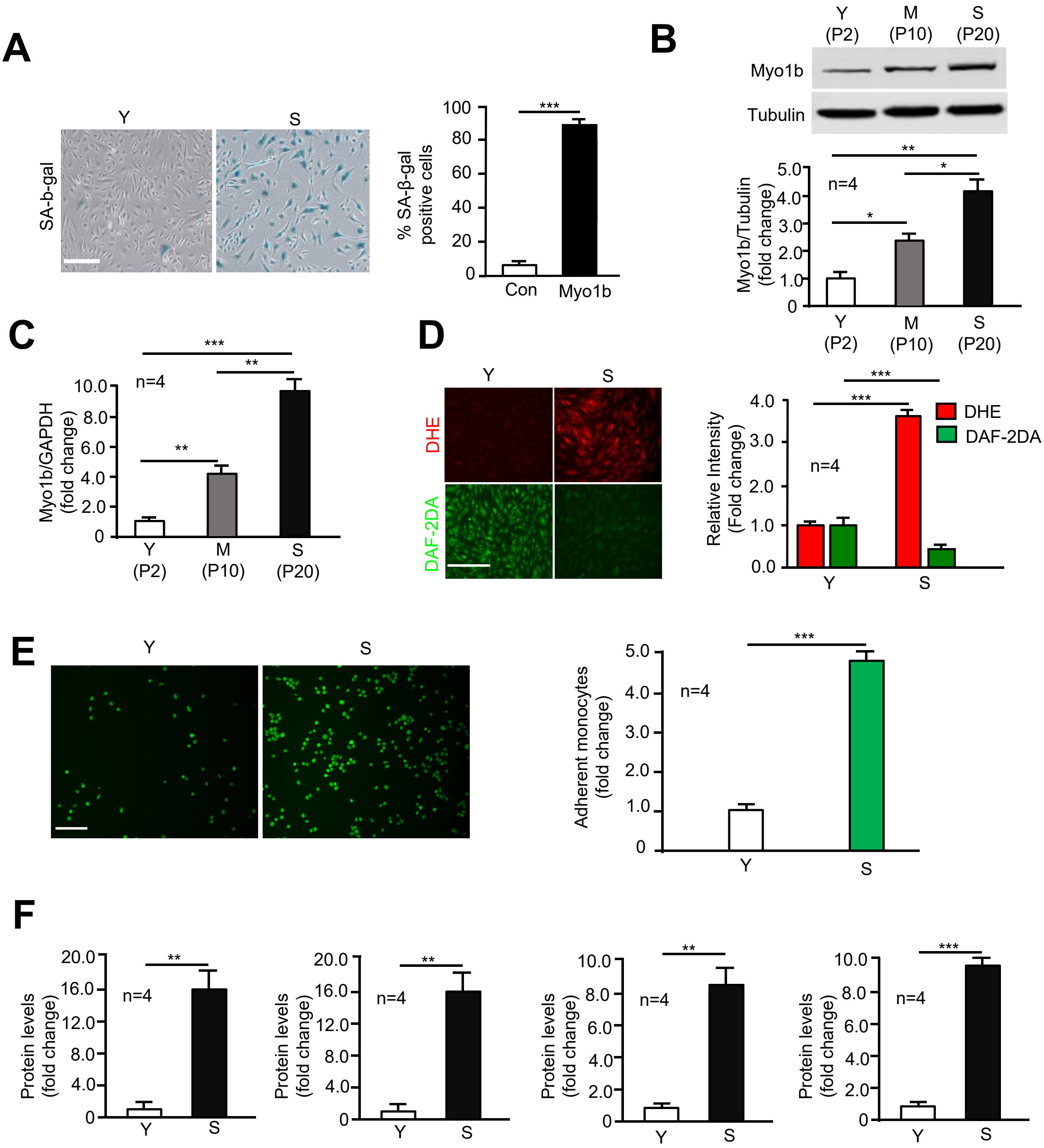

Supplementary Figure. Upregulated Myo1b and cytokine secretion in senescent endothelial cells with endothelial dysfunction. (A) SA-β-gal staining. Bar graphs on the right show quantifications of positive cells. Scale bar = 0.25 mm. (B) The protein and (C) mRNA levels of Myo1b in senescent endothelial cells. Bar graphs show quantifications of the markers. Tubulin served as loading control. (D) DHE and DAF-2DA staining for the detection of superoxide anion and NO. (E) THP-1 monocyte-HUVECs adhesion analysis. Bar graphs show quantifications of the adhered monocytes. (F) ELISA analysis of the secretion of IL-6, IL-8, TNF-α and MCP-1 from conditioned medium. (Y: young HUVEC, S: senescent HUVEC, n=4, * indicates $p< 0.05$, ** indicates $p< 0.01$, *** indicates $p< 0.001$).
